# Obstetrics

**Published:** 1876-10

**Authors:** 


### I. Obstetrics.

1. *Triple Pregnancy.* Leriche. (*Lyon Méd.*, July 9, 1876.)

A woman, in her third labor, was delivered of a male infant at 4:30 p. m., another at 5:30 p. m., and a female child at 6:30—the first presented by the vertex, the second by the breech, and the third by the shoulder. The first child seemed to be at term; the second had the development of a fœtus at eight and a half months; and the third of seven, or seven and a half months. All were vigorous and well formed. The placenta weighed 510 grammes. It was divided into two unequal masses, united by a common cotyledon. The smallest mass, which was symmetrically rounded, gave origin to an umbilical cord of ordinary volume, forty ctm. in length. This was attached to the first child. The other placental mass was larger and furnished two cords, each like the first having a distinct amniotic cavity. The smaller of these, thirty-six ctm. in length, was attached to the second child. The third, (corresponding to the third infant,) was slender and varicose, but thirty-three ctm. in length. It was inserted upon the external border of the placenta. The two cords which emerged from the same placental mass were attached to fœtus of a different sex.

